# Prevalence of Non-alcoholic fatty liver disease using fatty liver index (FLI) among health care workers - A cross sectional study

**DOI:** 10.6026/973206300212707

**Published:** 2025-08-31

**Authors:** Suriyaa Ravichandran, Nithin Theckumparampil, Ravindhra Bharathi Govindan, Meenakshi Vedavyasan

**Affiliations:** 1 Department of Biochemistry, Pondicherry Institute of Medical Sciences, Pondicherry, India; 2 Department of Radiodiagnosis, Pondicherry Institute of Medical Sciences, Pondicherry, India; 3 Department of Family Medicine, Pondicherry Institute of medical Sciences, Pondicherry, India

**Keywords:** Non-alcoholic fatty liver disease, Fatty Liver Index, Health care workers

## Abstract

Healthcare workers (HCWs) have faced increased risk of life-style related disorders such as non-alcoholic fatty liver disease (NAFLD) due to stress and workload following the COVID-19 pandemic. Therefore, it is of interest to evaluate the prevalence of NAFLD and risk factors among 184 Indian HCWs aged over 40 using a non-invasive Fatty Liver Index (FLI) as a diagnostic tool. NAFLD prevalence was 25.7% (95% CI: 19.8-32.5) using an FLI cut-off ≥60, with significant associations found for age, obesity, dyslipidemia, and metabolic syndrome. While no significant association was seen with HbA1c or sex, FLI showed fair agreement with abdominal ultrasonography (kappa = 0.31), supporting its potential as a practical cost-effective tool for large-scale screening rather than a confirmatory diagnostic tool. Given the growing NAFLD burden in high-risk occupational groups, FLI can enable early detection and targeted interventions in HCWs after establishing population-specific cut-off scores.

## Background:

Non-alcoholic fatty liver disease (NAFLD), the most common chronic liver disease, impacts over 25% of the population globally [1]. NAFLD, initially a benign condition, characterized by simple fat accumulation in the absence of alcoholism or secondary liver disease, can progress to inflammatory state known as Non-alcoholic steatohepatitis (NASH), which can advance further to fibrosis and hepatocellular carcinoma, a leading cause of liver transplants, which is beyond the reach of the low- and middle-income population [2]. It is emerging as a grave health threat, as its prevalence is projected to exceed 50% by 2040 in parallel to the rising metabolic risk factors, in the post-COVID-19 era, due to significant lifestyle changes [3]. Alarmingly, the prevalence of NAFLD in India ranges from 9% to 53%, with pooled average of 38.6% and remains a cause of concern [4]. Healthcare workers (HCWs), the backbone of the healthcare system are at increased risk of non-communicable disorders (NCDs) due to occupational stress, unhealthy lifestyles and an inadequate support system [5]. Particularly, they experienced tremendous stress and excessive work burden during COVID pandemic, a 21st century-catastrophe and many of them also battled and survived acute COVID-19 infection, while responding to the health crisis [6]. NAFLD, a multisystem disorder, has emerged as notable complication of long-COVID [7, 8]. Studies from China and Brazil have reported rising incidence of NAFLD among HCWs with prevalence rates above 30% [9, 10].

Unfortunately, limited attention has been given to NAFLD in HCWs in India, despite rising metabolic risk factors and critical need for an early diagnosis and intervention [11, 12]. Since the well-being of HCWs is vital for the effective functioning of healthcare system, an estimate of disease burden is essential for planning effective strategies and resources allocation [13]. Abdominal ultrasonography (USG) has been routinely used for the diagnosis of NAFLD, while the gold standard has been liver biopsy-an invasive tool with post-procedural complications [2]. The time involved in the procedure, need for clinical expertise, low sensitivity in detecting mild hepatosteatosis, particularly in obese patients, and inter-observer variability makes USG unsuitable for large-scale NAFLD screening and with NAFLD being asymptomatic many true cases of NAFLD can be missed allowing its progression to irreversible stage, where the treatment option is minimal [14]. This had initiated research to design alternative markers for early-stage detection of NAFLD which led to the emergence of various indices that combine both clinical features and biochemical parameters for NAFLD diagnosis [15]. The Fatty Liver Index (FLI) is an algorithm-based diagnostic tool developed by Bedoni *et al.* in 2006, specifically for Italian population incorporating four measured parameters: Body Mass Index (BMI), Waist circumference (WC), Serum Triglycerides (T) and Gamma-glutamyl transferase (GGT) [16]. Although FLI is widely validated across many populations, endorsed by international liver and diabetic associations for mass screening, it is yet be evaluated in Indian settings [17]. A review by Biciusca *et al.* on the role of FLI in NAFLD diagnosis and management highlighted variations in the distribution of risk factors across populations, warranting cohort studies to define local determinants and guide tailored management strategies [18]. Therefore, it is of interest to report the prevalence of NAFLD in Indian HCWs, using the non-invasive, cost-effective FLI, as an alternative to USG and to describe associated risk factors to guide targeted preventive interventions, particularly in low-resource settings, as there is a limited data on the metabolic risk burden in this occupational group.

## Methodology:

This cross-sectional study was undertaken as a part of the ICMR STS fellowship program, among the health care workers aged above 40 years who had enrolled for the annual health check-up programme in staff health clinic of a tertiary care hospital in Pondicherry. Pregnant women, individuals with pre-existing disease of the heart, kidney, liver, as well as those with chronic alcoholism and chronic infections, were excluded. Taking the prevalence of NAFLD as 25%, and confidence level as 95%, the required sample size was calculated as 184. Participants fulfilling the selection criteria and consenting were consecutively selected for study.

## Ethical considerations:

The Institutional Ethics Committee (IEC:RC/2022/161) gave its permission for this study. Informed consent was taken from the participants.

## Data Collection:

Socio-demographic, clinical and anthropometric details-including blood pressure, waist circumference (WC) and body mass index (BMI) were collected. Biochemical analysis for fasting glucose, lipid composition, liver function tests and HbA1c was performed using Rosche Cobas integra 400 biochemistry auto analyser and Bio-Rad D10 HbA1c analyser. Ultrasonography was performed by consultant radiologist using Siemens S2000 instrument. The NAFLD was diagnosed based on increased echogenicity compared to the right kidney parenchyma and was graded accordingly.

## Calculation of Fatty liver index (FLI):

The FLI was calculated using FLI calculator which uses the formula [17],

FLI = e^x/(1+e^x ) x100

Where x = 0.953 x InTGL+0.139 x BMI+0.718xInGGT+0.053 x WC-15.745 and TGL - Triglycerides (mg/dl), BMI - Body Mass Index (Kg/m^2^), GGT- Gamma glutamyl transferase (U/L), WC- Waist circumference (cm). A score of <30 rules out the disease and score of ≥ 60 indicates the presence of disease. A score of 30-60 is considered indeterminate.

## Evaluation of the metabolic syndrome is done according tonational cholesterol education program:

Adult Treatment Panel III (NCEP: ATPIII). Uric acid/HDL ratio (UHR) is calculated as a predictor of insulin resistance, metabolic syndrome and cardiovascular disease [19]. FLI was also compared with another non-invasive marker, hepatic steatosis index (HSI) [20].

## Hepatic steatosis index calculation:

=8*ALT / (AST)+BMI+2(If female)+2 (if diabetic)

## Interpretation:

HSI of <30 rules out NAFLD and ≥36 indicate a positive diagnosis for NAFLD.

## Statistical analysis:

Data were analysed using STATA version 18. Categorical variables were presented as frequency and percentages and continuous variables were expressed as Mean ± Standard Deviation (SD). Prevalence was calculated as a proportion of NAFLD-positive participants among those enrolled and evaluated in the study. The association between risk factors and NAFLD was estimated as prevalence odds ratio with 95% CI. The chi square test was used to determine statistical significance, and logistic regression analysis for risk factors of interest: Type2 diabetes mellitus (T2DM), hypertension, metabolic syndrome, dyslipidemia, UHR, family history of diabetes and obesity. Statistical significance was defined as p-value of less than 0.05. The agreement between USG-based diagnosis and FLI in the diagnosis of NAFLD was estimated using prevalence and bias adjusted kappa statistic with 95% CI. Similarly, agreement between FLI and HSI was also estimated.

## Results and Discussion:

This study is among the first in India to estimate the prevalence of NAFLD using FLI, specifically in HCW population residing in a semi-rural setting. An earlier Indian study used a variant of the FLI that included fasting insulin, which is not a routine test [21]. Our research design is akin to a prospective study carried out in Irish cohort in 2022 that used FLI for estimation of NAFLD prevalence and related risk factors [22]. The mean age of the study population was 48.1 ± 5.3 years, with a female majority (62%). The mean BMI of 26.8 ± 4.5 kg/m^2^ and mean waist circumference of 96.8 ± 9.8 cm indicate high prevalence of overweight and central obesity. 38.6% (n=71) had a low FLI score ruling out NAFLD, 35.3% (n=66) had FLI between 30-60, and 26.1% (n=47) had FLI ≥ 60. The prevalence of NAFLD was 25.7% (95% CI: 19.8-32.5), defined by FLI ≥60. This is lower than global and Indian estimates pooled prevalence (32%), but aligns with previous reports from HCW population of Brazil and India, notably it closely matches the NAFLD prevalence from Indian rural regions, similar to our semi-rural coastal population, undergoing urbanization [4, 10]. Biochemical analysis showed mean total cholesterol of 188.5 ± 35.5 mg/dl, triglycerides 121.3 ± 69.5 mg/dl, HDL 44.5 ± 9.3 mg/dl, and LDL 131.7 ± 32.7 mg/dl. The total cholesterol to HDL ratio and LDL to HDL ratio were 4.4 ± 1.1 and 3.08 ± 0.9, respectively- indicative of dyslipidemia. Non-HDL cholesterol was 144.1 ± 35.0 mg/dl, suggesting increased atherogenic risk. The mean HbA1c was 6.1 ± 1.5%, indicating prediabetes per ADA criteria. The mean uric acid to HDL ratio (UHR) was 11.1 ± 5.0, a surrogate marker linked to metabolic risk [19]. Table 1(see PDF) shows significant association between NAFLD and risk factors- increasing age, BMI, waist circumference, cholesterol/HDL ratio and metabolic syndrome (P<0.05). For each additional year of age, the odds of NAFLD increased by 6%. Sex had no association with NAFLD risk which aligns with findings in Indian and Irish cohorts. Age-related decline in metabolic and detoxifying functions of liver, along with rising metabolic risk predispose older adults to NAFLD [22]. Risk for NASH and HCC also increases with age, necessitating geriatric intervention [23]. The odds of NAFLD increase by 72% per unit rise in BMI and 22% per unit in WC underscoring the strong association with adiposity. Abdominal obesity promotes insulin resistance via inflammatory cytokines and leptin, accelerating fibrosis [24, 25]. Dyslipidemia showed a strong association with NAFLD, with odds increasing 62% per unit rise in cholesterol/HDL ratio. Notably, no significant association was found between NAFLD and diabetes. Metabolic syndrome was highly prevalent in the NAFLD group. Given the bidirectional relationship between metabolic syndrome and NAFLD, the observed association is expected and consistent with previous findings [24, 25]. The review by Biciusca *et al.* aligned with our findings on age, adiposity, dyslipidemia, and an elevated AST/ALT ratio (indirectly evaluated through HSI) as major risk factors for NAFLD, but diverged in reporting a higher prevalence among men, and stronger associations with T2DM, prediabetes, and hypertension, even in low-risk lean individuals [18]. This review is particularly relevant and a useful benchmark, as it examined NAFLD defined by FLI (≥60), similar to our approach, highlighting the need for population-specific evaluation of FLI in Indian HCWs with appropriate risk stratification.

The diagnostic accuracy of FLI score was evaluated against USG-based NAFLD reporting. The prevalence and bias-adjusted kappa is 0.36 (95%CI: 0.26- 0.46), indicating a fair agreement. The major disagreement was observed in the category of indeterminate (FLI: 31-60). The USG-based diagnosis of NAFLD showed high sensitivity (95.74%, CI: 85.46-99.48) but modest specificity (55.15%, CI: 6.39-63.68). FLI may serve as a screening tool rather than a confirmatory diagnostic test. FLI also showed fair agreement with another surrogate marker, HSI score with a bias adjusted kappa of 0.37(95% CI 0.24 - 0.49). As liver health is intricately linked to cardiovascular health, NAFLD is integrated into the Indian Government's National Program for Prevention and Control of Cancer, Diabetes, Cardiovascular Diseases and Stroke (NPCDCS) [2]. In this context, our study supports the utility of FLI as a simple and cost-effective tool for NAFLD screening and monitoring therapeutic response particularly in resource-restrained settings. Biciusca *et al.* shared a similar perspective, emphasizing FLI's primary value as a screening instrument for epidemiological studies rather than a definitive diagnostic test, citing its inability to grade disease severity, the ambiguity of intermediate scores (30-60), and reliance on cut-offs originally validated in European cohorts. Beyond its role in NAFLD, FLI also serves as a key "algorithm for diagnosis and prognosis of metabolic risk" with predictive role for diabetes, cardiovascular disease, and all-cause mortality. It can be considered an indispensable tool in NAFLD management, as reductions in FLI scores reflect improvements in BMI, waist circumference, and lipid profile after lifestyle modification, bariatric surgery, or pharmacotherapy-an aspect not assessed in our cross-sectional design [18]. With rising global NAFLD prevalence, specifically in Southeast Asia, there is an urgent need for screening, and FLI may play a pivotal role in population-based studies, especially in high metabolic risk groups. This is particularly relevant since even our medically informed HCW cohort demonstrated a high burden of metabolic risk factors, underscoring the need for targeted prevention.

## Conclusion:

FLI is a simple, practical tool that utilizes anthropometric and biochemical parameters and is free from limitations commonly associated with ultrasonography. The limitations of our study include a small sample size, lack of representation from the general and younger population, inability to assess disease severity and absence of data on lifestyle factors. With the rising burden of obesity and non-communicable diseases in the post-COVID era, there is an urgent need for large-scale screening using FLI, after establishing population specific cut-offs.
